# Performance Assessment of Carbon Dioxide Sequestration in Cement Composites with a Granulation Technique

**DOI:** 10.3390/ma17010053

**Published:** 2023-12-22

**Authors:** Jeong-Bae Lee, Jun-Hyeong Kim, Byeong-Gi Min, Byeong-Hun Woo

**Affiliations:** 1 Department of Civil Engineering, Daejin University, 1007 Hoguk-ro, Pocheon-si 11159, Republic of Korea; jblee@daejin.ac.kr; 2 Geomarble-Labs, 6-26, Jeonggeum-ro 162beon-gil, Gasan-myeon, Pocheon-si 11167, Republic of Korea; bspring@nate.com; 3 Civil and Environmental Engineering Department, Hanyang University, Jaesung Civil Engineering Building, 222 Wangsimni-ro, Seongdong Gu, Seoul 04763, Republic of Korea

**Keywords:** carbon dioxide, sequestration, granulation, coating, cement composites

## Abstract

The cement industry emits a significant amount of carbon dioxide (CO_2_). Therefore, the cement industry should recycle the emitted CO_2_. However, sequestration by carbonation in cement composites absorbs a very small amount of CO_2_. Therefore, a direct way of achieving this is to improve the absorption performance of CO_2_ in cement composites. In this study, to improve absorption, unlike in existing studies, a granulation technique was applied, and the material used was calcium hydroxide (CH). In addition, granulated CH was coated to prevent a reaction during the curing of cement paste. The coated CH granule (CCHG) was applied to 5% of the cement weight as an additive material, and the specimens were cured for 91 days to wait for the coating of CCHG to fully phase-change. The experiment of CO_2_ absorption showed an unexpected result, where the use of blast furnace slag (BFS) and fly ash (FA) had a negative effect on CO_2_ sequestration. This was because BFS and FA had a filler effect in the cement matrix, and the filler effect caused the blocking of the path of CO_2_. In addition, BFS and FA are well-known pozzolanic materials; the pozzolan reaction caused a reduction in the amount of CH because the pozzolan reaction consumed the CH to produce a calcium silicate hydrate. Therefore, the pozzolan reaction also had a negative effect on the CO_2_ sequestration performance combined with the filler effect. The CO_2_ sequestration efficiency was decreased between ordinary cement paste and BFS-applied specimens by 45.45%. In addition, compared to cases of ordinary cement paste and FA-applied specimens, the CO_2_ sequestration performance was decreased by 63.64%. Comprehensively, CO_2_ sequestration performance depends on the porosity and amount of CH.

## 1. Introduction

The cement industry emits a large amount of carbon dioxide (CO_2_). The amount of CO_2_ emission from the cement industry accounts for approximately 7% of global emissions and is the second largest source of carbon emissions worldwide [1]. Due to its large amount of CO_2_ emissions, the cement industry has a responsibility to reduce CO_2_. It is well known that cement composites react with CO_2_ and generate calcium carbonate (CC) following the process of Equation (1).

(1) $$Ca{\left(OH\right)}_{2}+CO_{2}\;\to CaCO_{3}+H_{2}O$$

Cement composites consume calcium hydroxide (CH) and generate the CC. With this process, cement composites absorb almost 70% of the CO_2_ that is generated by the production of cement during their service life [2]. However, this process takes a long time to absorb a sufficient amount of CO_2_ [2]. The earth is suffering from the effects of greenhouse gases, therefore, greenhouse gases need to be reduced, especially in the cement industry [3]. Therefore, the acceleration of CO_2_ absorption using cement composites with some special material needs to be studied.

Skocek et al. [4] focused on the mechanism of the reactions between hardened cement paste and CO_2_. The special point of Skocek et al. [4] was the use of recycled concrete. The results indicated that the carbonated paste from recycled concrete could be used as a supplementary cementitious material when the paste was ground into a fine powder [4]. In addition, the capturing performance of CO_2_ was increased, and the saving potential was 114.5 g of CO_2_ per 100 g of the carbonated paste [4]. A similar study was performed by Huntzinger et al. [5], who confirmed CO_2_ sequestration performance using cement kiln dust. The main components of the cement kiln dust were CaO and SiO_2_, and the minor components were MgO and K_2_O. As expected from the results of Huntzinger et al. [5], CaO was the main reactive component of CO_2_. However, contrary to their expectations based on the demonstrated factor of the CO_2_ reaction with MgO [6], the reaction between K_2_O and CO_2_ was larger than that of MgO and CO_2_ [5]. Taking into account the unexpected results from Huntzinger et al. [5], cement kiln dust could be used as a CO_2_-capturing material. Eloneva et al. [7] performed a CO_2_-fixing experiment using blast furnace slag. This experiment showed the potential of blast furnace slag as a suitable material for fixing CO_2_ [7]. On the other hand, a natural material was also studied as a sequestrating material for CO_2_: red mud [6]. The main component of the red mud was SiO_2_, and its content of CaO was almost 2 to 8%, which is a relatively smaller amount than the recycled material or byproduct [6]. Due to the composition of the red mud, it was expected that the CO_2_ capturing performance would not be good. However, Na_2_O also had a reactive characteristic with CO_2_, therefore, red mud had enough potential to capture CO_2_ [6].

Another method of using cementitious waste is to use a byproduct such as fly or bottom ash. In particular, the creation of municipal solid waste (MSW)-incinerated bottom ash (MSWIBA) is a problem that has been highlighted recently. Bangkok has increased MSW generation by 493%, Ha-Noi has increased it by 492%, Jakarta has increased it by 506%, and Wuhan has increased it by 600%, respectively, because of the COVID-19 pandemic [8]. In addition, MSWIBA cannot be reused, because the materials are incinerated [6] and it contains toxic heavy metals [9]. Therefore, MSWIBA is considered a material that is difficult to treat. However, Rendek et al. [10] found that MSWIBA could capture CO_2_. MSWIBA has a sufficient amount of CaO, MgO, and Na_2_O, therefore, these components react with CO_2_, and then the CO_2_ can be fixed [10]. In addition to bottom ash, lightweight fine aggregates can also be considered, like MSWIBA. Luo et al. [11] investigated CO_2_ absorption using zeolite. Since zeolite has pores, CO_2_ absorption was confirmed, and CH consumption was detected at the same time. In another case, Gupta et al. [12] investigated carbon sequestration performance using lightweight foamed cement mortar. Gupta et al. [12] added biochar particles as an additional material for the fine aggregate, rather than a replacement material. Biochar and the foamed cement matrix include a much higher ratio of pores than ordinary cement mortar, therefore, the CO_2_-passing paths were more abundant inside the hardened cement paste than in the ordinary case. However, the most important part is the application of materials. Making passing paths for CO_2_ is a good concept, however, an investigation from the perspective of materials should be the first priority. Comprehensively, the key component for capturing CO_2_ was CaO [6]. In addition, all existing studies only used the materials themselves, without any special treatment. In addition, those materials contained reactive components, therefore, the loss of materials occurred through reactions during cement hydration [4,13,14]. The purpose of CO_2_ capturing is to accelerate the reaction and maximize the sequestration efficiency. In order to achieve these conditions, it is important that the CO_2_ reactant survives in an unreacted state. Therefore, a study on this aspect should be carried out.

The investigation of CO_2_ movement paths was carried out by artificially increasing the internal spaces, but in no case was a general hardened cement paste product used. In addition, there were few cases in which additional methods for protecting reactants were considered. Therefore, it was judged that it is necessary to investigate the effect of the CO_2_ path through a general hardened cement paste product in a general state, and to study improvement in CO_2_ sequencing performance through the coating protection of CH granules. This study focused on using CH as a CO_2_ reactant and maximizing CO_2_ capturing performance, while preventing this material from reacting with cement as much as possible using granulation and coating techniques. Through this technique, it was expected that the CO_2_ sequestration performance of cement composites would be increased remarkably.

## 2. Materials and Experiments

### 2.1. Materials

The binder was the ordinary Portland cement (OPC), defined in the ASTM C150 [15]. The OPC which was used in this study is a type I cement whose composition can be easily found in other studies, and is listed in Table 1 [16,17,18].

**Figure 1:**
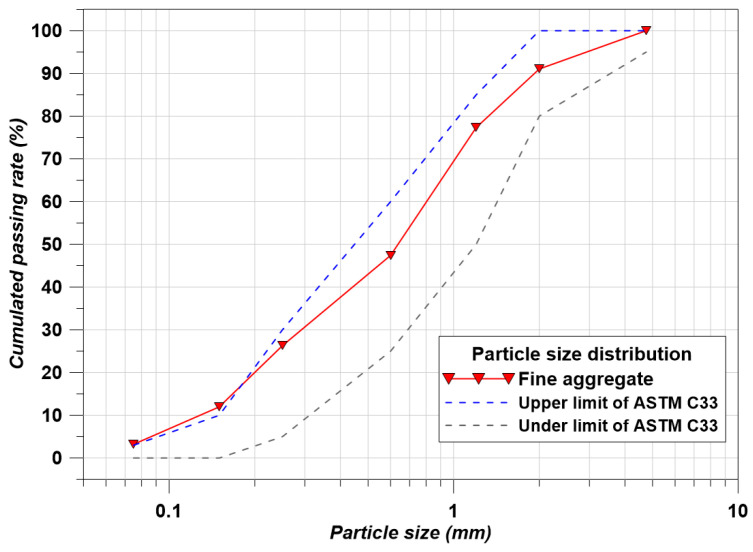
PSDs of fine aggregate.

Natural sand was used as the fine aggregate, with a fineness modulus of 2.55, a density of 2.63 ton/m^3^, a water absorption of 1.35%, and particle size distribution (PSD) as shown in Figure 1 [19].

**Figure 2:**
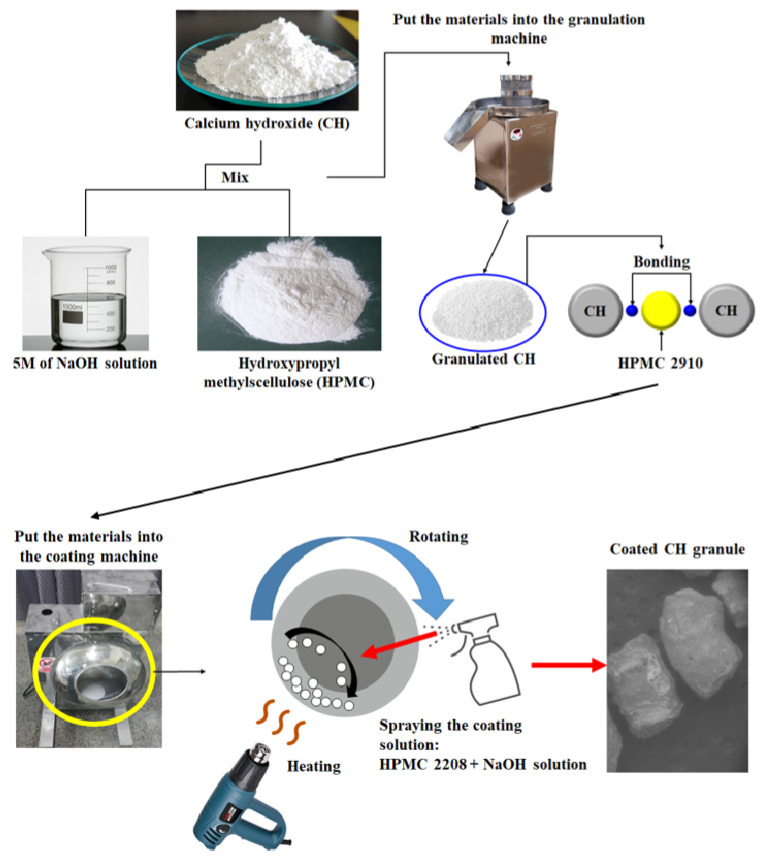
The process of making the coated CH granules.

In this study, to accelerate CO_2_ capturing, CH was used as a granule material. Because CO_2_ reacts with the calcium in the cement matrix [20], the CH was considered the most suitable material for CO_2_ capturing. To granulate the CH, a binder was needed to bond the CH particles, and the material chosen was hydroxypropyl methylcellulose (HPMC), HPMC 2910. In addition, to prevent the reaction between the OPC and granulated CH, a coating process was added, and the coating material was a hydrophilic material, HPMC 2208. Since the coating should be molten in order for the granulated CH to react with CO_2_, HPMC 2208 was a suitable choice for the coating material. Here, HPMC numbers (2910 and 2208) refer to the grade of HPMC. Its details are indicated in Table 2, and the method of this study is presented in Figure 2.

**Figure 3:**
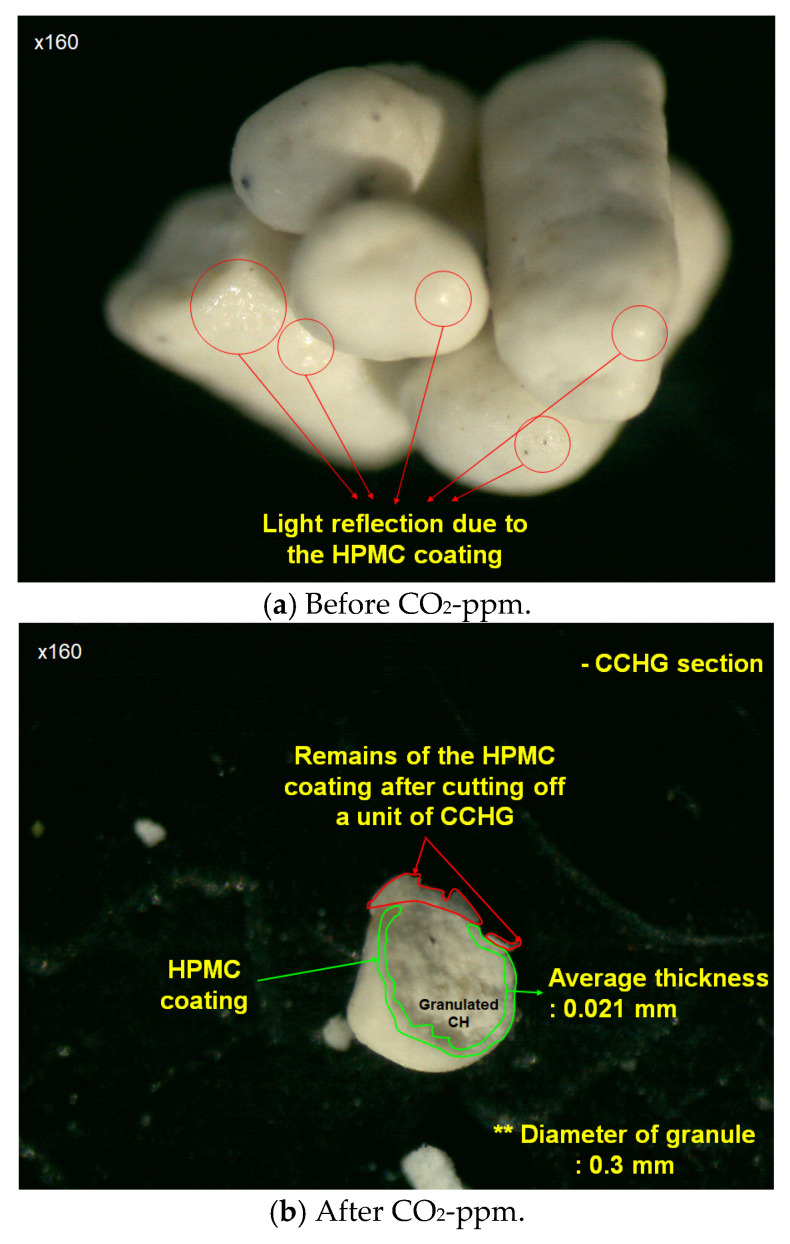
Coated CH granules.

The coated CH granules (CCHG), as shown in Figure 3, were used as an additive material, and the dosage ratio was 5% of the cement weight. The reason for the 5% dosage was that the HPMC is an organic material, and organic material usually decreases the mechanical performance of cement composites [21,22,23]. Because of this negative effect, the dosage of organic additive had to be less than 5% of the cement weight [22] or water weight [21]. Therefore, a dosage ratio of 5% of the cement weight was chosen, considering both strength performance and CO_2_ sequestration performance. The mix’s details are presented in Table 3.

Additionally, commonly used by-products, which were fly ash (FA) and blast furnace slag (BFS), were used. It is already well known that FA and BFS improve the durability of cement composites [24,25]. Because the pozzolanic components cause the pozzolan effect [26,27] and the particle size of FA and BFS causes the packing effect [28], most of the performance factors of the cement composites (including durability) improve.

BFS has sufficient potential for the sequestration of CO_2_ [29,30], since BFS has a sufficient amount of CaO, as shown in Table 4. In addition, the CO_2_-capturing performance of the FA shows that FA also has enough potential for the sequestration of CO_2_ [31], the composition of FA is shown in Table 4.

### 2.2. Experiment

**Figure 4:**
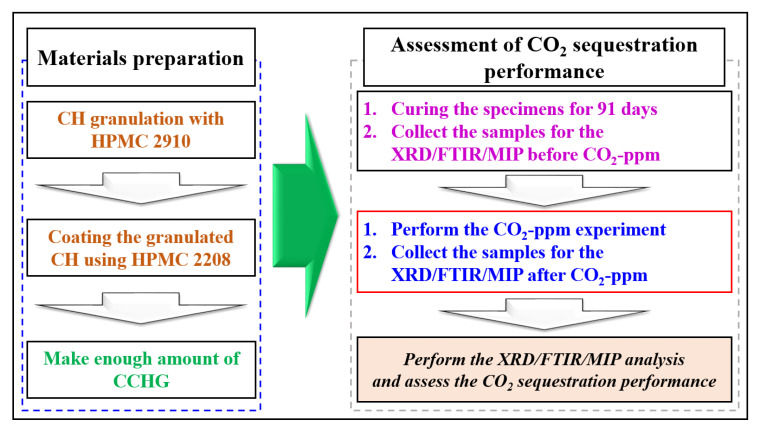
Comprehensive process of the experiment.

To investigate the CO_2_ sequestration performance, CO_2_ sequestration monitoring was carried out by ppm (CO_2_-ppm), X-ray diffraction (XRD), mercury intrusion porosimetry (MIP), and Fourier transform infra-red (FTIR). All the specimens were cured for 91 days in the air, with 65% relative humidity and 20 °C air temperature. The reason for the long duration of curing was that we intended to wait for the coated material to undergo a full phase-change. Due to the long duration of curing, components such as ettringite disappeared, and only core components like CSH, portlandite, and calcite remained. Therefore, the analysis was simplified due to the long-term curing. The comprehensive process of the experiment is presented in Figure 4.

#### 2.2.1. CO_2_ Sequestration Monitoring by ppm (CO_2_-ppm)

**Figure 5:**
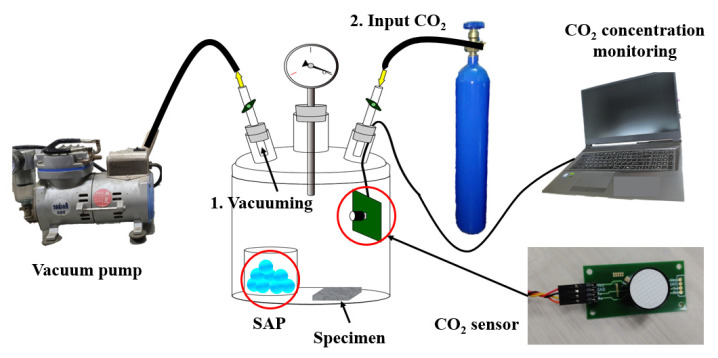
CO_2_ concentration control.

**Figure 6:**
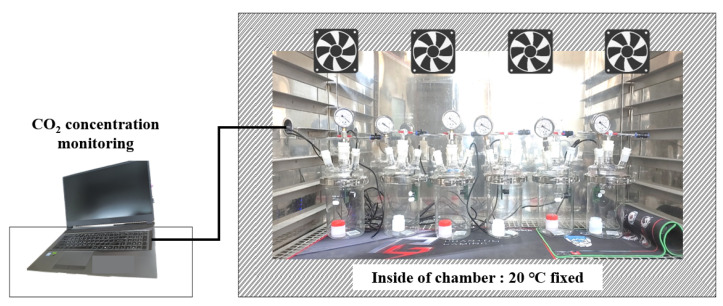
CO_2_ sequestration experiment.

To specifically assess the CO_2_ sequestration performance, some factors were controlled. The concentration of CO_2_ was controlled at 99.99% in the atmosphere because the concentration of CO_2_ in the air is quite low. The CO_2_ concentration controlling system is indicated in Figure 5. To bring the concentration of CO_2_ to 100% in the desiccator, the inside of desiccator featured a specifically made vacuum. After vacuuming the inside of the desiccator, the air flowing pipe at the side linked with the vacuum pump was closed, and the pipe at the side linked with the CO_2_ tank was opened. The inside of the desiccator was completely filled with CO_2_ from the CO_2_ tank, and the injection status of CO_2_ was monitored using the air pressure gauge installed in the desiccator. In addition, the CO_2_ concentration was checked using the CO_2_ sensor (Econarae Co., Yongin-Si, Republic of Korea, Accuarcy—99% ± 0.5%) installed in the desiccator. While the CO_2_ reacts with CCHG, water is generated, and the generated water makes the sensing accuracy decrease, because the CO_2_ sensor is sensitive to humidity. Thus, a super absorbent polymer (SAP) was placed in the desiccator to absorb the generated water. Including the CO_2_ controlling system, the full experimental system is shown in Figure 6.

Cement composites are a kind of heterogeneous material. Thus, their temperature should be fixed, because fine aggregate and CCHG particles are randomly placed in the cement matrix. Randomly placed materials can not be controlled, therefore, it is essential to fix the outside temperature. The desiccators containing the specimens were placed in the temperature-controlled chamber, and the room temperature of the chamber was fixed to 20 °C for a measuring duration of 5 days.

#### 2.2.2. X-ray Diffraction (XRD)

The reaction between CCHG and CO_2_ was confirmed by crystallization, and the results were confirmed by the XRD. The XRD specifics were 40 kV, 3 mA, a scanning speed of 2°/min, and a wavelength of 1.54 Å (D/max2500, Rigaku Corporation, Tokyo, Japan). All the specimens’ crystalline components were measured by XRD before CO_2_-ppm and after CO_2_-ppm. Through these comparisons, the reaction of CCHG could be confirmed.

#### 2.2.3. Fourier Transform Infra-Red (FTIR)

The crystalline production could be confirmed by XRD, however, we could not sufficiently demonstrate the sequestration performance with only XRD. For the exact evaluation of CO_2_ sequestration performance, supporting analyses were needed, and FTIR (Nicolet 6700, Thermo Scientific, Waltham, MA, USA) was one of them. Through the FTIR, the reaction details between CO_2_ and CCHG were analyzed.

#### 2.2.4. Mercury Intrusion Porosimetry (MIP)

The distinctive characteristic of this study was that the matrix of specimens was denser than before the CO_2_-ppm experiment. However, this general assumption could have been made before the experiment was carried out, therefore, we needed to demonstrate how much the porosity decreased. In the MIP test (Micromeritics Autopore IV 9500, Micromeritics Instrument Co., Norcross, GA, USA), the pore size could be calculated by Equation (2):

(2) $$\gamma =\frac{-2\delta cos\theta }{p}$$

where γ is the radius of pore (μm), δ is the mercury surface tension (N/m, with 0.484 N/m applied), θ is the contact angle between the specimen and mercury (with 130° applied), and p is the input pressure of mercury (with 206.84 MPa applied). Following the pressure, the measurable pore size was 3 nm.

#### 2.2.5. Thermogravimetric Analysis (TGA)

CO_2_-ppm was the intuitive method chosen for showing that the CO_2_ sequestration performance, and TGA (TGA7 PERKIN ELMER, TA Instruments, New Castle, DE, USA) was the quantitative method chosen for showing that the trend of the chemical analysis was right. The temperature range was 25 to 900 °C. CH and CC were mainly checked, and in particular, the increments in CC before and after the CO_2_-ppm experiment.

## 3. Results and Discussion

### 3.1. CO_2_-ppm Results with MIP

**Figure 7:**
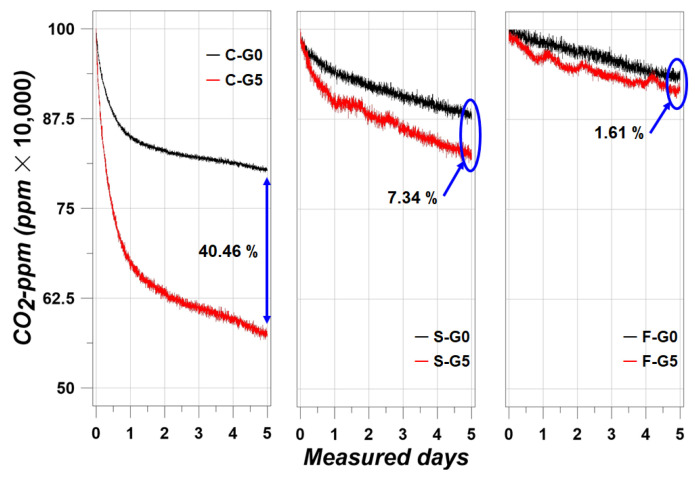
CO_2_-ppm results.

The CO_2_-ppm trends were clear. S-G0 and S-G5 were expected to show the best performance in CO_2_ sequestration due to their CaO contents with BFS and cement. However, according to Figure 7, it was unexpected that the C-G0 and C-G5 would show the best sequestration performance.

**Figure 8:**
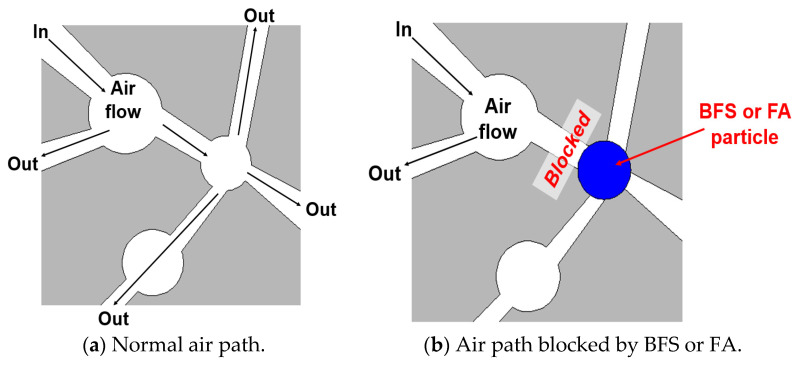
The case of normal and blocked air paths in the cement matrix.

There was a large gap in the absorbed amount of CO_2_ compared to C-G0, C-G5, and the other specimens. In the case of C-G5, the absorbed amount of CO_2_ was increased to 40.46% compared to C-G0. C-G0 absorbed the CO_2_ at about 198,000 ppm, and C-G5 absorbed the CO_2_ at about 429,000 ppm. This was evidence that the CCHG significantly improved the CO_2_ sequestration. However, the other specimens showed a relatively poor sequestration performance compared with C-G0 and C-G5. It was considered that the filler effect from BFS and FA disturbed the improvement in CO_2_ sequestration performance. As is well known, BFS and FA make cement composites denser [32,33]. This means that the porosity is reduced, in other words, the path of airflow is also reduced. This concept can be explained roughly by Figure 8.

**Figure 9:**
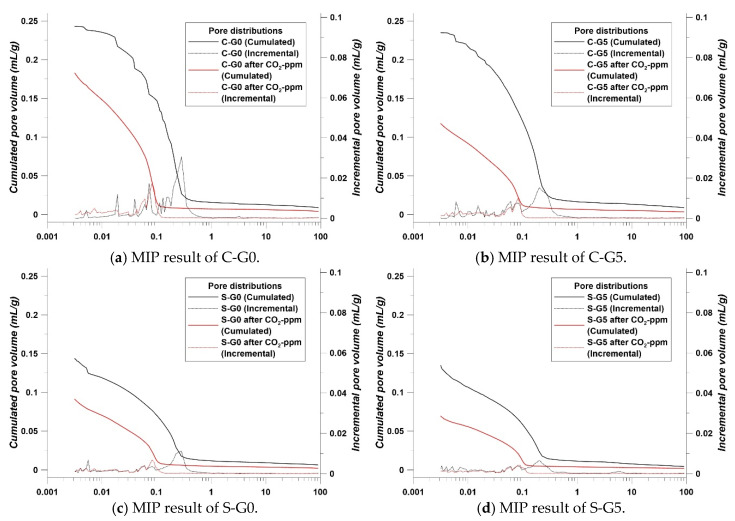

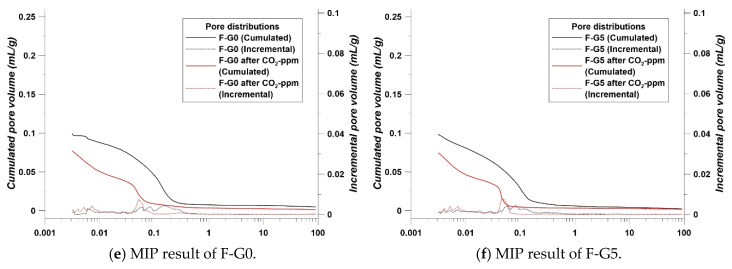
MIP results.

The concept of Figure 8 is directly related to air permeability, and it has been reported that BFS and FA reduce air permeability significantly [34]. When the air path was blocked by BFS or FA particles, the CO_2_ flow was also significantly disturbed. Evidence for the concept of Figure 8 is in the porosity trends shown in Figure 9.

Like the trends reported in [32,33,34], the porosity results showed that S g series and F g series showed less porosity than C g series. In addition, CCHG also brought about a minimal filler effect, as confirmed by Figure 9. Overall, the reason that the CO_2_-ppm results appeared as in Figure 7 can be understood using Figure 8 and Figure 9.

The S g and F g series showed a strong packing phenomenon, as shown in Figure 9. Within this phenomenon, it was considered that the blocking of air flow in the cement matrix was reduced significantly by the BFS and FA particles. Most studies on the carbonation of cement composites have focused on preventing or reducing carbonation depth [35]. Therefore, the results in Figure 9 are commonly understood. However, interestingly, the use of BFS or FA brought about a negative effect on absorbing CO_2_ in this study. Due to the packing effect shown in Figure 8, CO_2_ could not flow in the cement matrix, therefore, CO_2_ could not touch the CCHG. Then, the CCHG could not sequestrate the CO_2_, and the CO_2_-ppm efficiency was significantly decreased by the packing effect.

Paradoxically, contrary to what is well-known, the use of BFS and FA led to a decrease in CO_2_ sequestration performance by reducing the porosity and blocking the path of CO_2_. Here, a new factor was found: a higher porosity had a good effect on improving the CO_2_ sequestration performance of the cement composites.

### 3.2. XRD Resutls

**Figure 10:**
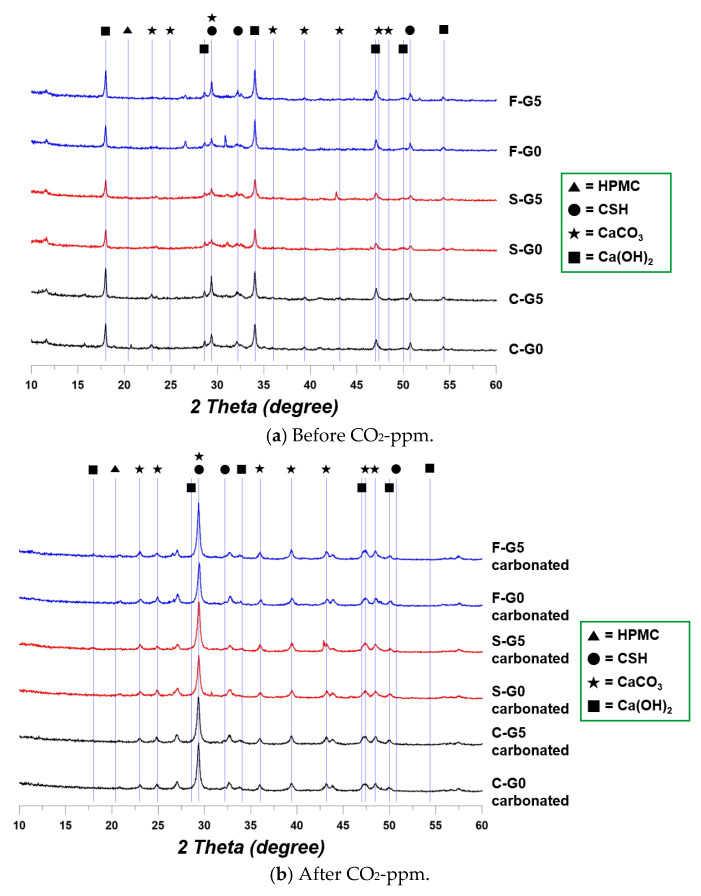
XRD results.

According to the XRD data in Figure 10, calcite was detected in all the cases after CO_2_-ppm. The theta degrees of 17.98, 34.10, 47.12, 50.81 indicate portlandite, and it can be confirmed that portlandite clearly disappeared, especially at the theta degree of 17.98 in the G0 case. These results indicate that carbonation proceeded normally [36]. However, an interesting point was that portlandite was detected in all the G5 specimens after CO_2_-ppm at the theta degree of 47.12 and 50.81. This phenomenon appeared when the portlandite remained after carbonation, in this case, the intensity at the theta degree of 17.98 was very weakly detected, or not detected at all [37]. However, at theta degrees of 47.12 and 50.81, the remaining portlandite could be detected [37]. Therefore, portlandite points were detected after CO_2_-ppm in the cases of G5 specimens.

From the XRD results, it was demonstrated that the CCHG absorbed the CO_2_ enough, and the CCHG produced calcite after reacting with CO_2_. In addition, the HPMC fully disappeared due to the long-term curing.

### 3.3. FTIR Results

**Figure 11:**
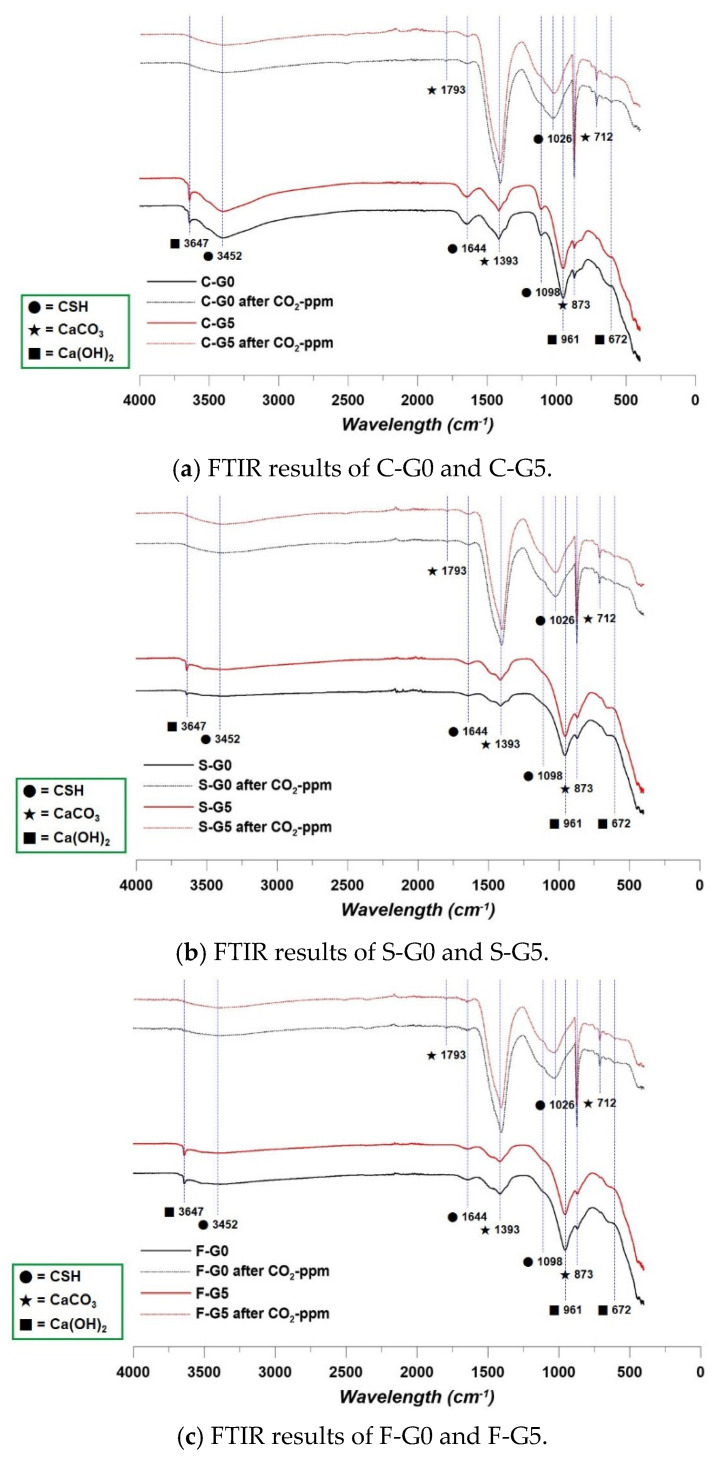
FTIR results.

The FTIR results showed a remarkable difference between before and after CO_2_-ppm. The most distinctive difference was the calcite. As in the XRD results, the wavelength absorption was significantly increased at the calcite points in all specimens. The FTIR results are indicated in Figure 11.

Due to the long-term curing, the portlandite intensities were different. The curing duration was enough time for the pozzolanic reaction. Therefore, the intensity points of portlandite in the F g and S g series were relatively more weakly detected than in the C g series [38]. At the characteristic 3452 wavelength point in the cases of F g and S g specimens, the intensity was increased after CO_2_-ppm, because the BFS and FA consumed the CH, and it made the CSH [38]. In addition, all the cases showed the intensity of CSH was weakened, not indicating a reduction in CSH, but instead indicating an increase in calcite. 

All the data from CO_2_-ppm, MIP, XRD, and FTIR indicated an increase in calcite after the reaction of CCHG and CO_2_. In addition, due to the pozzolanic reaction, CH was consumed, and this led to a reduction in the CO_2_ sequestration performance, because CH was the key component in consuming CO_2_.

### 3.4. TGA Results

**Figure 12:**
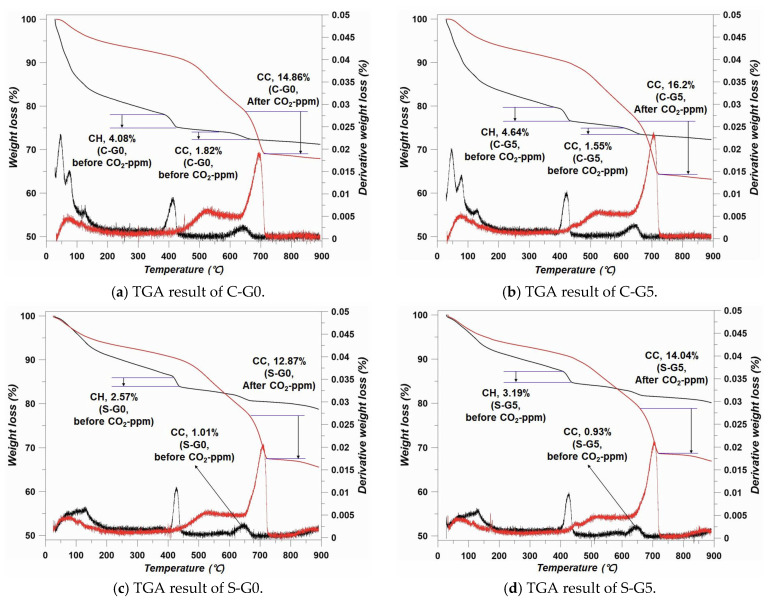

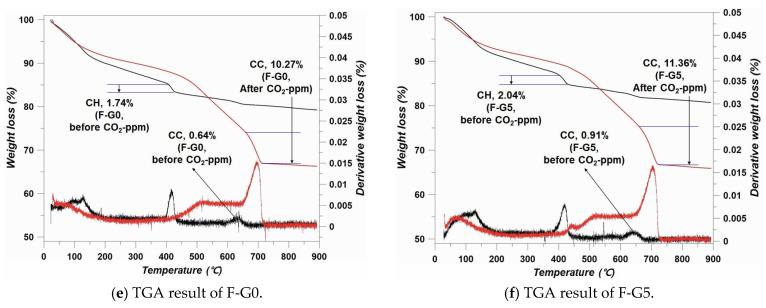
TGA results.

TGA results are presented in Figure 12. The distinct difference was the detected absolute amount of CC. Usually, the CH appears around 420 °C, and the CC appears in the range of 550 to 740 °C [39,40]. The amount of CC before/after CO_2_-ppm showed the same trend as the XRD and FTIR results. The intensity of the CH disappeared after CO_2_-ppm according to the XRD and FTIR, but the intensity of CC increased in all cases. Showing the same trend, the amount of CC increased in the TGA data after the CO_2_-ppm. Compared to the MIP results, the absolute amount of CC after CO_2_-ppm proved that the pores in the cement matrix were important to sequestrate the CO_2_. Due to the filler effect, as mentioned in the MIP chapter, the moving path of CO_2_ was blocked or narrowed by the FA and BFS. Therefore, the increment in and absolute amount of CC tended to decrease after CO_2_-ppm, as shown in Figure 12. In addition, the CH peaks almost disappeared in all specimens after CO_2_-ppm, this meant that the peaks in XRD and FTIR showed the components accurately.

### 3.5. Summary Discussion

The performance improvement in CO_2_ sequestration with CCHG was demonstrated by the CO_2_-ppm. In addition, it was focused on obtaining the reliability of CO_2_ sequestration performance through a component analysis by XRD and FTIR, and porosity confirmation by MIP. Before carrying out the CO_2_-ppm, the problem was that the coated HPMC was fully phase-changed. This point was confirmed by the XRD patterns. The peak of HPMC appeared nearby the theta degree of 20 [41], and the peak of HPMC was not detected in all the cases of G5 specimens. Therefore, it can be considered that the coated HPMC was fully phase-changed.

The CO_2_-ppm experiment results were unexpected, because existing studies have reported that the BFS and FA are helpful to sequestrate the CO_2_ [42,43]. Of course, those existing studies treated the materials to meet the aim of their studies. However, the usual cases use the FA or BFS as a supplementary material to the cement, therefore, the packing effect of FA and BFS [32,33,34] had a negative effect on the CO_2_ sequestration in this study. The packing effect was confirmed by the MIP results, which showed that the porosity was significantly reduced than C-G0 and C-G5 before CO_2_-ppm. In addition, the pozzolanic reaction was also confirmed due to the long-term curing. The pozzolan reaction consumes the CH to make the CSH, therefore, the amount of CH was reduced in the cases of F g and S g specimens, and it led to the reduction of CO_2_ sequestration performance.

Comprehensively, to sequestrate the CO_2_ enough, higher porosity was helpful in capturing the CO_2,_ paradoxically. This is because the packing effect caused the blocking of the path of air flow, and it disturbed the reaction between CCHG and CO_2_. However, high porosity caused a reduction in the mechanical strength [44]. Therefore, the concept of this study should not be applied to main structures such as columns, slabs, and beams, because this concept needs high porosity, and thus may not have enough strength. Thus, this concept is suitable for substructures that are not burdened by external loads, such as the median strip on roads.

One limitation of this study is the use of a lot of CH powders to make granules. The procedure for making CH powders needs high temperatures, like the production of cement. High production temperature definitely produces CO_2_. Hence, further works should use other materials that have high reactivity with CO_2_ and low production temperatures with the method of this study to overcome this limitation.

## 4. Conclusions

This study focused on improving the CO_2_ sequestration performance of cement composites using a granulation technique. The main material was CH, and the binder of CH was HPMC 2910. The granulated CH should not be in the reactive state, therefore, it needed a coating to prevent the reaction between cement paste and granulated CH while the cement composites were curing. The granules were coated with HPMC 2208 and the specimens with CCHG were cured for 91 days to wait for the coating to fully phase-change. Our comprehensive conclusions are as follows:

1. The CO_2_-ppm results were contrary to the expectation that the F g and S g groups would show better performance than the C g group. This is because the BFS and FA caused the filler effect, and this phenomenon blocked the path of CO_2_ flow as result. Therefore, CCHG could not react with CO_2_ enough because of the BFS and FA. In short, BFS and FA had a negative effect on the CO_2_ sequestration performance.
2. According to the XRD, FTIR, and TGA results, one other negative factor was found: the pozzolan effect caused the consumption of CH. As is well known, the pozzolan reaction consumes CH to make CSH. However, CH is a key component in reacting with CO_2_ in the cement matrix, therefore, the amount of CH was decreased by the pozzolan reaction during the curing period. The CH consumption was found in the results of XRD, FTIR, and TGA. Due to this phenomenon, combined with the filler effect, the sequestration performance was significantly decreased.
3. As a comprehensive result, CO_2_ sequestration depends on the porosity and the amount of CH in the cement matrix. For this reason, the matrix did not have enough strength, therefore, the concept of this study is suitable for substructures are not burdened by external loads, such as the median strip on roads.

## Figures and Tables

**Table 1:** Properties of OPC.

Chemical Composition (%)					
SiO_2_	Al_2_O_3_	Fe_2_O_3_	CaO	MgO	Other
20.8	6.3	3.2	62	3.3	4.4
**Physical Properties**					
**Blaine (cm^2^/g)**			**Specific Gravity (ton/m^3^)**		
3200			3.15		

**Table 2:** Details of the granulation materials.

Type	Details of Materials
Granule	Main composition: CH (95%) Diameter: 0.3 mm, Length: 0.4~0.6 mm
Granule binder	Main composition: HPMC with grade 2910 (HPMC 2910) Viscosity: 3 mPa·s HPMC-to-solvent ratio: 1%
Coating material	Main composition: HPMC with grade 2208 (HPMC 2208) Viscosity: 100,000 mPa·s HPMC-to-solvent ratio: 0.25% Coating method: rotating pan coating
Solvent	5 M of NaOH solution

**Table 3:** Mix details.

Specimens	Mix Ratio (On the Basis of Cement)					
	Water	Cement	Sand	CCHG	Fly Ash	BFS ^1^
C-G0	0.485	1	2.75	0	0	0
F-G0					0.1	0
S-G0					0	0.1
C-G5				0.05	0	0
F-G5					0.1	0
S-G5					0	0.1

^1^ BFS = blast furnace slag.

**Table 4:** Chemical composition of FA and BFS.

Materials	SiO_2_	Al_2_O_3_	Fe_2_O_3_	CaO	LOI
FA	56.4	23.2	7.6	3.3	1.0
BFS	34.5	16.1	0.6	42.6	0.05

## Data Availability

Data are contained within the article.
